# Agonistic Behavior Is Affected by Memory in the Dusky Damselfish *Stegastes fuscus*

**DOI:** 10.3389/fnbeh.2021.663423

**Published:** 2021-08-17

**Authors:** M. M. Silveira, J. F. de Souza, H. Araujo-Silva, A. C. Luchiari

**Affiliations:** Departamento de Fisiologia e Comportamento, Centro de Biociências, Universidade Federal do Rio Grande do Norte, Natal, Brazil

**Keywords:** aggression, individual recognition, social interaction, cognition, reef fish

## Abstract

The ability to discriminate familiar from unfamiliar conspecifics has been demonstrated in several species of fish. Agonistic interactions are among the most frequent behaviors exhibited by territorial species and could offer useful information for the individual recognition process. In agonistic situations, memory may modulate the behavioral response and affect social dynamics, but few studies have explored the memory retention acquired during aggressive encounters. The present study investigated the memory retention of an agonistic encounter in the dusky damselfish *Stegastes fuscus*. The experimental procedure was divided into three parts: (1) Familiarization; (2) Recognition test; and (3) Memory test. During the familiarization phase, the fish were visually exposed to the same conspecific for 5 days (10 min per day) and the behavior was recorded. On the following day (conspecific recognition test), half of the animals were paired with the same conspecific and the other half with a different conspecific for 10 min, and the behavior was recorded. The fish were retested 5, 10, and 15 days after the test to evaluate memory retention. In the memory test, they were exposed to the same conspecific as before or to a different conspecific. We found that the damselfish reduced their agonistic displays when the stimulus fish was familiar, but when it was unfamiliar, the animals were more aggressive and only reduced their mnemonic response after 10 days. These results suggest that the recognition ability of damselfish can be affected by time and that it modulates agonistic response.

## Introduction

Recognition, a complex process required in most social interactions (Tibbetts and Dale, 2007), has been demonstrated by many vertebrates (Beer, 1971; Bee and Gerhardt, 2002; Petrulis et al., 2004; Saeki et al., 2018). This ability requires visual (Dasser, 1987; Kohda et al., 2015), olfactory (Watanabe and Mori, 1990), and auditory sensory systems (Myrberg and Riggio, 1985; Balshine-Earn and Lotem, 1998; O’Loghlen and Beecher, 1999). Recognition occurs when one individual identifies another based on distinctive traits (Dale et al., 2001), usually changing its behavioral response (Tibbetts and Dale, 2007). Class-level recognition occurs when animals recognize groups, while specific-level recognition involves animals’ identifying specific individuals (Wiley, 2013). In any case, individuals need social interactions in which the exchange of relevant information can be used as a cue or signal to recognize an individual in a future encounter.

Not all species are able to recognize individuals, possibly due to their context or environmental pressures (Wiley, 2013). Some animals can recognize individuals after interacting with them (Wiley, 2013), which has been investigated in many vertebrate species, including fish. The effects of familiarity on fish have been demonstrated in social contexts such as breeding (Hert, 1985), courting (Dzieweczynski et al., 2012), shoaling (Chivers et al., 1995; Griffiths and Magurran, 1997; Barber and Wright, 2001; Sikkel and Fuller, 2010), social learning (Swaney et al., 2001), fear contagion (Silva et al., 2019) and especially agonistic behavior (O’Connor et al., 2000; Doran et al., 2019).

In territorial contexts, recognizing individuals and reacting accordingly could reduce defense-related costs. In addition, the dear enemy effect can be established, where the frequency and intensity of aggression are reduced in interactions with familiar animals (Fisher, 1954; Briefer et al., 2008). This phenomenon has been confirmed in territorial fish by the lower number of displays (Earley et al., 2003; Sogawa et al., 2016; Silveira et al., 2020) and changes in the use of space (Frostman and Sherman, 2004; Saeki et al., 2018) between neighbors. As a consequence, these agonistic interactions may have community-level impacts (Fontoura et al., 2020), and could provide useful information for the individual’s recognition process (Miklósi et al., 1992).

Although the role of learning and memory in the recognition process is well known, they have not been explored in detail for most species (Wiley, 2013). The memory of agonistic situations may modulate the behavioral response and affect social dynamics, but few authors have investigated memory retention acquired during aggressive encounters (Francis, 1983). Some studies have demonstrated different responses related to memory duration when the opponent is a conspecific or a heterospecific (Miklósi et al., 1992), suggesting that exposure time affects memory response (Csányi et al., 1989). Memory retention may be associated with the recurrence of the situation, where remembering is essential for decision making (Dall et al., 2005; Brown and Chivers, 2006). For instance, it may not be worth remembering a single encounter that would never be repeated (Miklósi et al., 1992), while recurring, or more significant experiences (i.e., an encounter with a predator) could be worthwhile (Mackney and Hughes, 1995). In the present study, we investigated the memory retention of an agonistic encounter in the dusky damselfish, *Stegastes fuscus*.

Damselfish, an important fish family that inhabits coral reefs, includes more than 340 species (Frédérich et al., 2009). These fish exhibit varying levels of highly agonistic behavior. Most defend their territories against co- and heterospecific intruders (Taylor and Francis, 2016), and may respond differently to confrontations involving familiar and unfamiliar individuals (Thresher, 1979; Silveira et al., 2020). According to a recent study that empirically investigated the agonistic interactions of reef fish, damselfish are one of the most important groups responsible for establishing coral reef communities (Fontoura et al., 2020). While most of our knowledge of reef fish ecology is based on damselfish, their learning and memory processes are poorly understood. Thus, there is significant interest in studies involving their cognitive processes. In the present study, we aimed at testing the effects of familiarity on memory retention time in the dusky damselfish *S. fuscus*. We hypothesized that agonistic memory would be affected by familiarization and that fish would remember their conspecific and behave less aggressively towards it. To that end, we compared the response between groups paired with familiar and unfamiliar opponents at three different times (5, 10, and 15 days) in order to determine memory retention time. We predicted that if fish could recognize each other, their aggression level would be higher in confrontations with unfamiliar individuals, while recognition would attenuate it.

## Materials and Methods

### Animal Collection and Husbandry

Adult dusky damselfish (*S. fuscus*) were collected from tide pools at Búzios Beach, Parnamirim, Rio Grande do Norte state, Brazil, using a cast net (3 m diameter, 10 mm mesh size), as authorized by the Brazilian Institute of the Environment and Renewable Natural Resources (IBAMA License Number 54688–1). The animals were immediately stored in 30-L containers with seawater and air stones to maintain oxygen level, and transported to the laboratory, where they were kept in a closed system with water recirculation for 15 days before the experiments in order to reduce the capture stress level. The system water was prepared with artificial salt and filtered water (35.2 g salt 1-l of water), kept at a temperature of 26–28°C, salinity of 35–38, and pH of 7.8. Thirty percent of the system water was exchanged every 7 days, except during the experimental phase, to avoid handling stress. The walls and bottom of the tanks were covered with gravel-wallpaper to increase well-being, and the dark-light cycle was set at 12 h:12 h in the experimental room. Each individual was maintained in an area with the same dimensions (25 × 40 × 30 cm). The fish were visually isolated to avoid confrontation stress but shared chemical cues because of water recirculation. They were fed three times a day, alternating between dry flakes (Sera Marin^®^ GVG-Mix47.7% protein) and *Artemia salina*. One hundred and sixteen fish were used, and all the experimental procedures were authorized by the Animal Ethics Committee of the Universidade Federal do Rio Grande do Norte (CEUA 041/2016).

### Conspecific Recognition and Memory

The experimental phase was conducted in the same tanks used for acclimation and maintenance in order to reduce handling stress. Each tank was divided in half by a glass wall covered with a removable partition covered with gravel-wallpaper. The animals could see each other after the partition was removed, but there was no physical contact. A pair of fish with similar weight and length were kept in each tank, one on each side.

**Figure 1:**
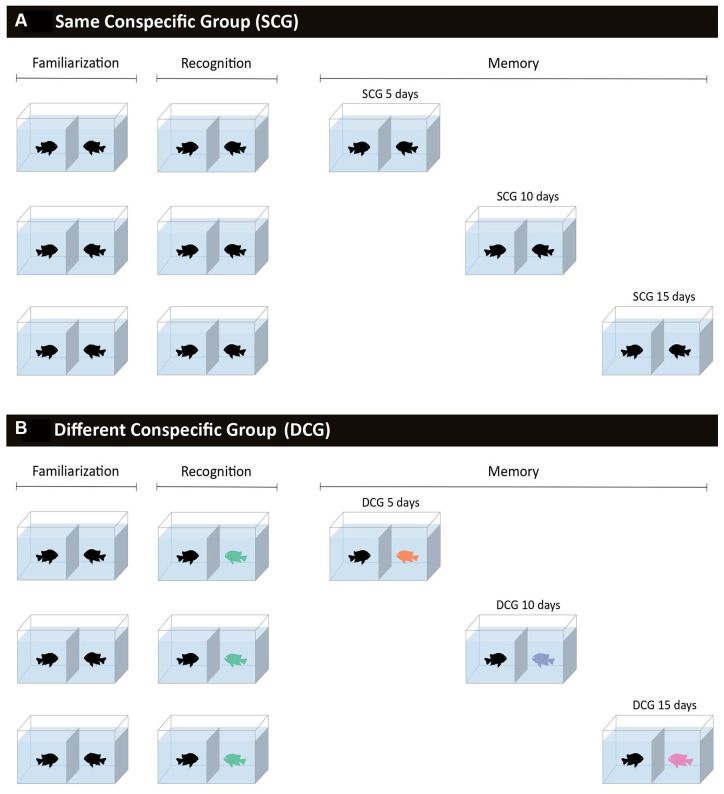
Schematic overview of the experimental procedure. Fish were divided into pairs and separated by a glass wall and a removable partition with gravel wallpaper that permitted visual contact when removed, for 10 min a day. **(A)** Same conspecific groups, in which fish were paired with the same conspecific during familiarization (5 days), recognition test, and memory tests (5, 10, or 15 days after the recognition test). **(B)** Different conspecific groups, in which fish were paired with one conspecific during familiarization and the conspecific was replaced by an unfamiliar individual in the recognition test and another in each of the memory tests (5, 10, or 15 days after the recognition test).

To test the effects of familiarity on memory retention time in damselfish, the experimental procedure was divided into three phases: (1) Familiarization; (2) Recognition test; and (3) Memory test (Figure 1). The familiarization phase (1) took 5 days, during which the wallpaper partition between the fish was removed, allowing them to see each other for 10 min once a day. This phase was the same for all fish pairs. Fifty-eight pairs of damselfish were tested. Behavior was recorded between 09:00 and 11:00, always before the fish were fed. Eight fish pairs were recorded at the same time with four cameras placed 1 m in front of the tanks (DVR Ch HDMI + Cam infra Ccd SONY^®^). Each camera captured two fish pair behaviors at the same time. The recording was repeated until all fish pairs were tested.

The recognition test (2) was applied the day after the familiarization phase. For this test, half of the fish pairs faced the same opponent as in the familiarization phase (29 pairs); repeating the same procedure as before for these animals (same conspecific group: SCG). However, the other half (29 pairs) faced an unfamiliar opponent (not previously paired) and could interact with a different fish (different conspecific group: DCG). The unfamiliar fish had gone through the same familiarization phase but faced another fish in the recognition test. Each encounter lasted 10 min and behavior was recorded as described above.

After the recognition test, the SCG and DCG fish were divided into three groups and submitted to the memory test at 5, 10, and 15 days (3). The pairs had no visual contact for the entire 15 days. For the SCG, the same fish pairs from the familiarization and recognition phases were tested after 5 (10 pairs), 10 (11 pairs), and 15 days (8 pairs). For the DCG, fish were always paired with an unfamiliar conspecific after 5 (10 pairs), 10 (11 pairs), and 15 days (8 pairs). At every encounter, fish pairs were initially separated by a wallpaper partition and after its removal, had visual contact with each other, and behavior was recorded for 10 min.

Thus, six groups were independently tested: the SCG_5_ with a memory interval of 5 days (*n* = 10), SCG_10_ 10 days (*n* = 11), SCG_15_ 15 days (*n* = 8), DCG_5_ 5 days (*n* = 10), DCG_10_ 10 days (*n* = 11), and DCG_15_ 15 days (*n* = 8).

Behavioral records were analyzed by two blind observers who registered the number of agonistic displays emitted during the confrontations and latency to the first attack. The behavioral inventory of *Stegastes fuscus*, focusing especially on aggressiveness, has been studied in a natural environment (Menegatti et al., 2003; Aued, 2012; Silveira et al., 2020) and under laboratory conditions (Silveira et al., 2015, 2019, 2020). When *S. fuscus* are allowed to interact, several types of aggressive behavior are observed, namely chasing, waving, tail or lateral biting, side or frontal confrontation, and bilateral, side or medial attacks (for description see Silveira et al., 2020), but when they are prevented from physical interaction by a glass window, aggressive behavior is reduced to fast approaches and quickly and successively attempting to bite their opponent (by biting the glass). Additionally, when displaying aggressive behavior, damselfish maintain their dorsal fin erect, anal fins expanded and their body color becomes darker. This type of display is well documented and was used as a response parameter to measure aggressiveness in previous studies with *S. fuscus* (Silveira et al., 2015; Da Silva-Pinto et al., 2020). Damselfish videos were analyzed and one blind observer counted the number of aggressive behaviors described above and the latency to the first attack.

For locomotor analysis, we used the ANY-maze Video Tracking System 6.33 (64-bit) (Stoelting Co., USA) to assess the following parameters: average speed while moving, total distance traveled, and time immobile. To analyze the time spent in different areas of the tank and the proximity of the animals to the stimulus fish, the tank was virtually divided into three vertical areas (proximal, intermediate, and distal) and fish occupancy of the areas was evaluated using the ANY-maze system.

### Statistical Analysis

All data were analyzed for homogeneity, normality, and possible outliers, as suggested by Zuur et al. (2010). Data from the familiarization phase (5 days) were grouped and shown as the average of the 5 days. Two-way ANOVA was conducted considering as factors “phases” (familiarization, recognition test and memory test at 5, 10, and 15 days) and “groups” (SCG and DCG), as well as the interactions between them, in order to test intergroup differences in the following parameters: total agonistic displays, latency to the first attack, average speed while moving, total distance traveled, and time immobile. When needed, Bonferroni *post hoc* tests were performed.

To compare time spent in each area of the tank, we also conducted two-way ANOVA considering “phases” and “areas of the tank” as qualitative variables. In case of a significant main effect (*p* ≤ 0.05), the Bonferroni *post hoc* test was performed when applicable.

Data were analyzed using “tidyverse”, “rstatix”, and “ggpubr” packages (Wickham et al., 2019; Kassambara, 2020a,b) in the RStudio program (RStudio, 2014). We considered an alpha level of 0.05 and 0.01 for statistical significance.

## Results

**Figure 2:**
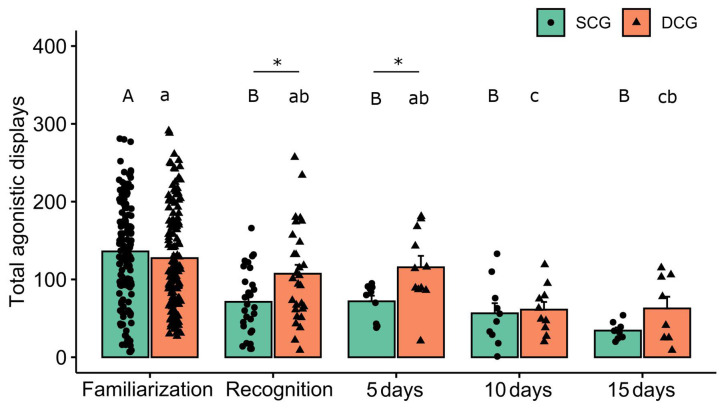
Total agonistic displays in each experimental phase. Bars (Mean + SE) indicate total agonistic displays of the dusky damselfish *Stegastes fuscus* for 10 min. Different uppercase letters indicate statistical significance in the SCG (two-way ANOVA, *p* < 0.05). Different lowercase letters indicate statistical significance in the DCG (two-way ANOVA, *p* < 0.05). An asterisk indicates a statistical difference between the SCG and DCG, in the same experimental phase (*p* < 0.05). *Post hoc* showed statistical significance between the SCG and DCG and SCG_5_ and DCG_5_ (*p* = 0.01) during the recognition test (*p* = 0.01).

Total agonistic displays, measured by the number of attacks by fish from the same (SCG) and different conspecific groups (DCG) are shown in Figure 2. Two-way ANOVA revealed no significant group effect (*F*_(1,389)_ = 0.18 *p* = 0.66), but showed statistical significance for phases (*F*_(4,389)_ = 17.03 *p* < 0.001) and interaction terms “groups vs. phases” (*F*_(4,389)_ = 2.42 *p* = 0.04). The Bonferroni *post hoc* test showed that total agonistic displays were higher during the familiarization phase and lower during the ensuing SCG encounters. For different conspecific groups, DCG_10_ and DCG_15_ differed in the familiarization phase, while DCG_10_ and DCG_5_ differed in the recognition test. The Bonferroni *post hoc* test also showed that in the familiarization experimental phase, SCG differed from DCG and SCG_5_ from DCG_5_ (*p* < 0.05).

**Figure 3:**
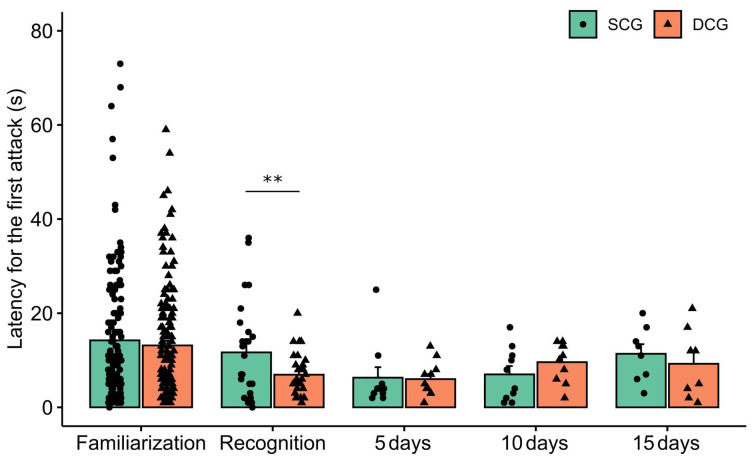
Latency to the first attack in each experimental phase. Bars (Mean + SE) indicate latency to the first attack of damselfish *Stegastes fuscus*. Two-way ANOVA was performed and the asterisk indicates a significant value in the comparison between the SCG and DCG during the recognition experimental phase (two-way ANOVA; *p* = 0.003).

Figure 3 depicts latency to the first attack in the SCG and DCG. The two-way ANOVA test showed statistical significance for phases (*F*_(4,389)_ = 4.07 *p* = 0.003), but none for groups (*F*_(1,389)_ = 1.45 *p* = 0.22) nor interaction between groups and phases (*F*_(4,389)_ = 0.46 *p* = 0.72). The Bonferroni *post hoc* test showed that the SCG differed from the DCG in the recognition test (*p* < 0.01).

**Figure 4:**
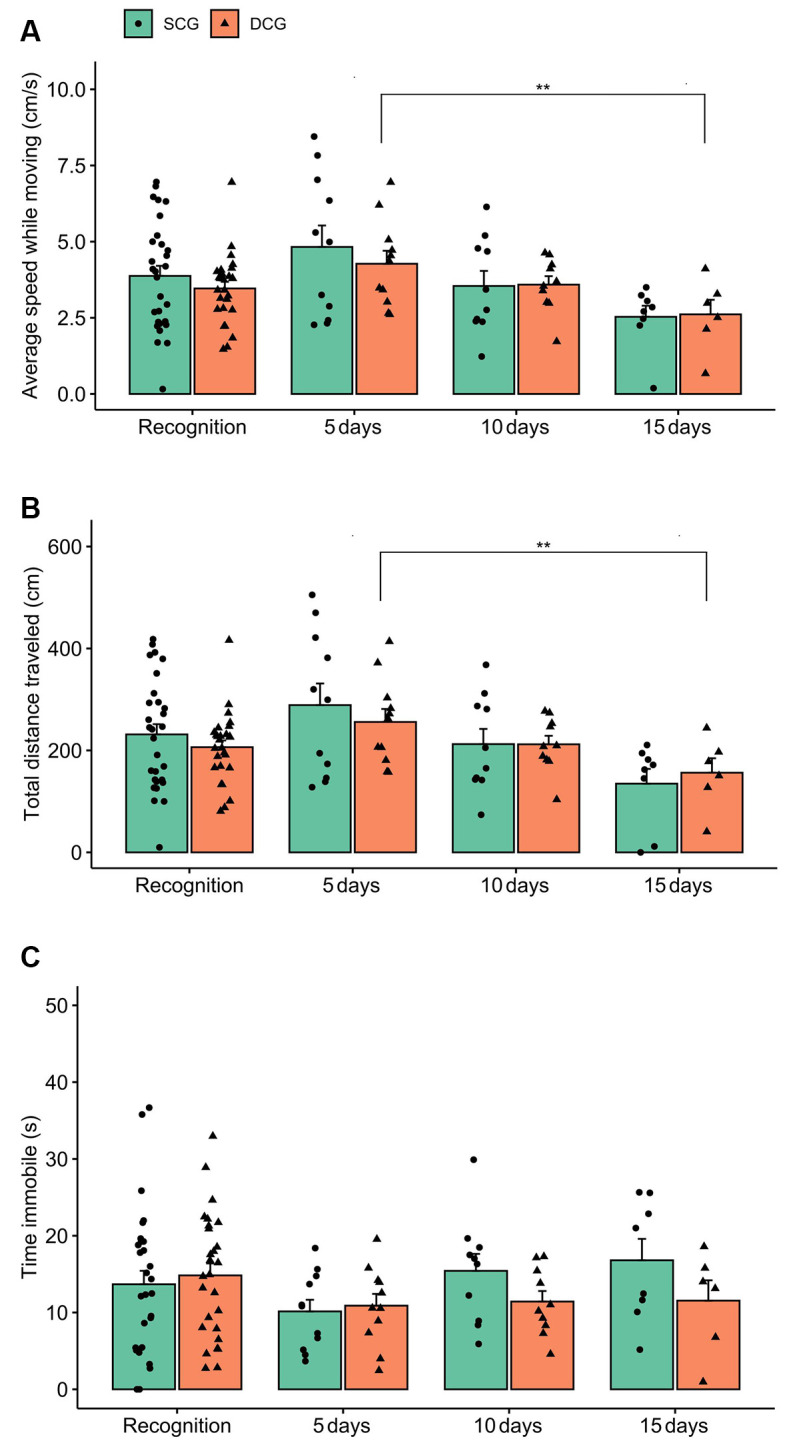
Locomotor behavior comparison between the SCG and DCG. Bars indicate dusky damselfish *Stegastes fuscus* locomotor behavior (Mean + SE) for 10 min in the test tank: **(A)** average speed while moving; **(B)** total distance traveled, and **(C)** time immobile. Two-way ANOVA was performed and the asterisk indicates a significant value in SCG and DCG comparison during the recognition experimental phase (two-way ANOVA; *p* = 0.003). Time immobile was not statistically significant between groups.

Figure 4 shows the locomotor parameters (average speed while moving, total distance traveled, and time immobile) of damselfish from the SCG and DCG. Two-way ANOVA exhibited no statistical significance in average speed while moving between groups (*F*_(1,104)_ = 1.06 *p* = 0.30) or in the interaction between groups and phases (*F*_(3,104)_ = 0.23 *p* = 0.87), but showed statistical significance for phases (*F*_(3,104)_ = 5.02 *p* = 0.003). The Bonferroni test indicated that DCG_5_ and DCG_15_ differed from each other (*p* < 0.01; Figure 4A). For total distance traveled, two-way ANOVA showed a significant phase effect (*F*_(3,104)_ = 5.60 *p* = 0.001), but no statistical significance for groups (*F*_(3,104)_ = 0.88 *p* = 0.35) or interaction terms group vs. phase (*F*_(3,104)_ = 0.34 *p* = 0.79). The Bonferroni test showed that DCG_5_ and DCG_15_ differed from each other (*p* < 0.01; Figure 4B). For time immobile, two-way ANOVA revealed no statistical significance for groups (*F*_(3,104)_ = 0.19 *p* = 0.65), phases (*F*_(3,104)_ = 1.38 *p* = 0.25), or interaction terms groups vs. phases (*F*_(3,104)_ = 1.07 *p* = 0.36; Figure 4C).

**Figure 5:**
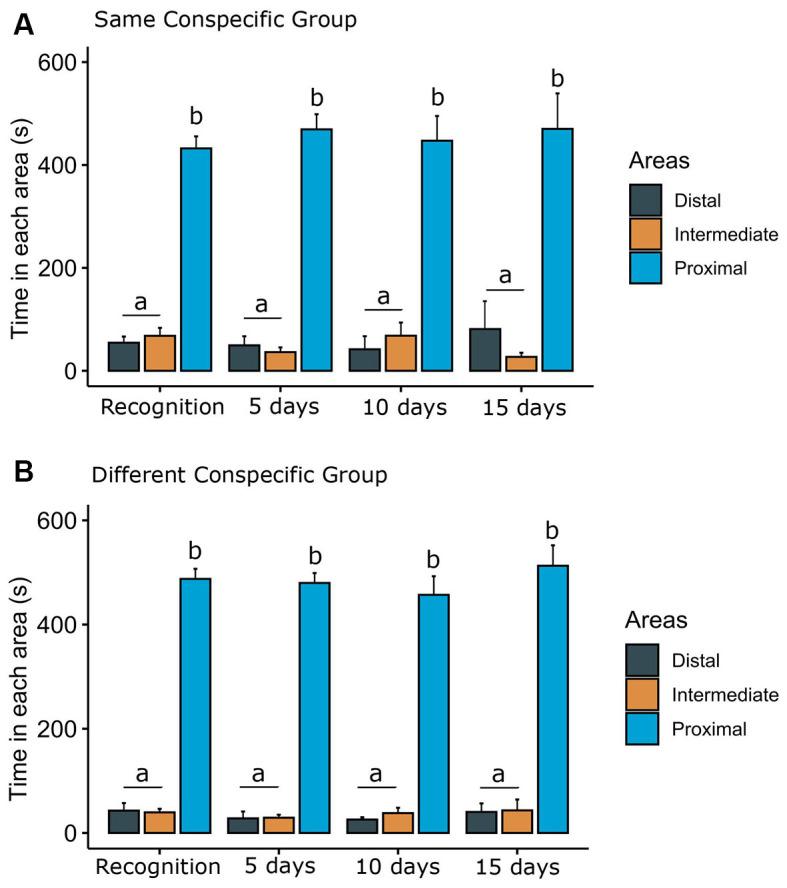
Time spent in each area of the tank. Bars (Mean + SE) indicate the time that the dusky damselfish *Stegastes fuscus* spent in each area of the tank for **(A)** the same conspecific group or **(B)** different conspecific group. The tank was divided into three virtual areas (proximal, intermediate, and distal). Two-way ANOVA showed that both animals visually exposed to the same and different conspecifics spent more time in the proximal area: different lower case letters indicate statistical significance (*p* = 0.001).

We also analyzed the time each focal fish spent in each of the three areas of the tank (Figure 5). For the SCG (Figure 5A), two-way ANOVA revealed statistical significance for areas of the tank (*F*_(2,162)_ = 267.70, *p* < 0.001), but no significant effect of phases (*F*_(3,162)_ = 0.03, *p* = 0.99) or interaction terms phases vs. areas (*F*_(6,162)_ = 0.58, *p* = 0.70). For the DCG (Figure 5B), two-way ANOVA demonstrated statistical significance for areas (*F*_(2,150)_ = 771.00, *p* < 0.001), but no effects of phases (*F*_(3,150)_ = 0.75, *p* = 0.58) or interaction between phases vs. areas (*F*_(6,150)_ = 0.23, *p* = 0.96). The Bonferroni test showed that animals visually exposed to the same and different conspecifics spent more time in the proximal area of the conspecifics.

## Discussion

We observed that familiarity between conspecifics affects the progression of agonistic displays in different phases of the experimental procedure. Damselfish exhibited a time-dependent reduction in the number of agonistic displays against familiar conspecifics compared to their unfamiliar counterparts. For the SCG, the decline in aggressiveness occurred immediately after the familiarization phase, while for the DCG fewer displays were observed after 10 days. This response suggests that the breaking point for a decline occurs 10 days after the previous encounter, but does not persist after 15 days. Thus, the identity of the intruder influences *S. fuscus’s* agonistic response and indicates that the animals recognize each other, but this ability appears to be affected time.

We also showed that animals spent more time close to the stimulus fish during the encounters, indicating that the vigilance behavior was preserved and was not influenced by familiarity or time. In regard to locomotor parameters, fish faced with familiar and unfamiliar conspecifics showed similar average speed, total distance traveled, and time immobile. This response demonstrates that all fish explored the tank and that differences in agonistic displays were not related to locomotion.

Aggression level was an important parameter in studies involving recognition and awareness of other individual’s identity in agonistic and territorial contexts. Familiarity with the opponent reduced aggressiveness in territorial fish (Thresher, 1978; Sogawa et al., 2016), a response consistent with the dear enemy phenomenon (Leiser and Itzkowitz, 1999; Frostman and Sherman, 2004; Sogawa and Kohda, 2018). In the present study, we compared the behavioral response of fish paired with familiar and unfamiliar opponents. A higher agonistic level was observed in unfamiliar pairs, suggesting that when animals do not recognize the stimulus fish, they maintain increased levels of signaling and attacks against the potential intruder. According to Saeki et al. (2018), who investigated TIR (True Individual Recognition) in territorial cichlid fish, the aggression level varies during a confrontation, exhibiting a higher number and stronger types of aggressive displays (i.e., bites) when the animals perceive the threat and react against it, while aggression decreases gradually over time.

By contrast, there was no variation in several aggressive displays during *S. fuscus* encounters, indicating that the behavioral response pattern was balanced during the 10-minute confrontation. This result may have occurred because *S. fuscus* is considered a very aggressive species that vigorously defends its territory and displays increased vigilant behavior (Osório et al., 2006; Silveira et al., 2020), which explains the preference for the proximal area of the tank. In our study, fish pairs were kept in the tanks (separated by an opaque partition) throughout the experiment and both focal and stimulus fish may have established territories. As such, both familiar and unfamiliar pairs were defending an area, which may have affected the number of displays during the visual confrontation.

With respect to the opponent’s memory, the SCG showed fewer aggressive displays immediately after the familiarization phase, persisting in the ensuing encounters, while the DCG maintained highly aggressive behavior after 5 days, but no differences were found between DCG_10_ and DCG_15_ time points. This suggests that the breaking point for a decline occurred after 10 days and no further decrease was observed after 15 days. *S. fuscus* seems to recognize and remember the stimulus fish up to 5 days after the previous encounter. This result suggests that *S. fuscus’s* visual memory of an opponent is retained for 5 days, and other factors may affect retention time, such as sensory inputs and physical interaction. In the present study, since animals could only access their opponents through a glass window, the fish had restricted information to identify the conspecific. Silveira et al. (2019) investigated memory retention of appetitive and aversive conditioning tasks and observed that, in both contexts, *S. fuscus* could learn and store the information for up to 15 days. While these authors tested a different protocol to evaluate memory, it may indicate that the nature of the task and type of stimulus influence the memory retention period. Comparable results were reported by Warburton and Hughes (2011), suggesting that memory windows could be different between species and situations. This hypothesis is supported by studies involving African cichlid groups that investigated long-term memory in different contexts using similar species. Ingraham et al. (2016) showed that *Labidochromis caeruleus* learn a food reward task and form a reversible discrimination memory for at least 12 days. However, according to Hotta et al. (2014), memory extinction can be anticipated in an agonistic confrontation context in *Julidochromis transcriptus*. These authors observed that losers’ memory is extinct 7 days after the encounter, who henceforth do not react against winners and other rivals as they did for 3–5 days of the confrontation.

It is known that the hippocampus (and analogous areas) plays an important role in declarative memory, spatial processing, and social recognition. This brain region may also be crucial for social cognition and context-dependent decision-making (Rubin et al., 2014). While it is accepted that memories are essential for survival in most animals, knowledge about the nature of long–term memory in fish remains limited (Laland et al., 2003; Ingraham et al., 2016). Some studies involving different species and contexts have shown conflicting results regarding the time intervals of mnemonic responses. In regard to food reward tasks, Williams et al. (2002) demonstrated that zebrafish can recall information involved in spatial alternation tasks after 10 days. Nilsson et al. (2008) trained Atlantic cod using an appetitive trace-conditioning paradigm and observed that they recall the stimulus for at least 3 months after the training phase. With respect to spatial learning abilities, Schluessel and Bleckmann (2012) investigated memory retention in gray bamboo sharks and observed that they retained the response in the absence of reinforcement for 6 weeks. In the natural environment, Triki and Bshary (2020) recently reported that *Labroides dimidiatus* exhibit a mnemonic response 11 months after an aversive event. These studies and ours suggest that environmental contexts, nature of the task, training protocol, and type of stimulus used significantly affect memory formation, retention, and extinction.

Although our knowledge of fish memory and the brain systems guiding memory formation is limited, some studies have identified the role of dopamine and glutamate in the mnemonic response of fish. Sison and Gerlai (2011) showed that the glutamatergic synapses need to be functional immediately after associative memory formation in zebrafish (*Danio rerio*) and Hamilton et al. (2017) identified the influence of dopamine receptors on memory formation in Caribbean bicolor damselfish (*Stegastes partitus*). Dopamine plays several important roles in behavior regulation, such as rewarding social stimuli and motivated behavior (Trainor, 2011; Riters, 2012; Weitekamp and Hofmann, 2017). The involvement of dopaminergic receptors in memory consolidation was also demonstrated by Naderi et al. (2016) in a study involving an associative learning task in zebrafish. Dopamine is known to be related to motivation, an important component in learning and memory processes (Warburton and Hughes, 2011).

Several studies have demonstrated that the learning and memory capabilities of teleost fish are as complex as those of mammals and birds, contradicting the idea that fish are less complex animals, with essentially instinctive cognitive abilities (Laland et al., 2003; Salas et al., 2006; Oliveira et al., 2015). In regard to agonistic/social memory, only a few studies have addressed this issue, which needs further investigation. For instance, Miklósi et al. (1992) observed that the memory of paradise fish opponents was detectable 1 day after the encounter, but the response disappeared 1 week later. For *S. fuscus*, the opponent was recognized after 5 days, but a number of gaps need to be filled for a thorough understanding of this species’ motivation for territorial defense and how interindividual recognition affects behavior. The present study contributes to the topic, showing that visual confrontations between territorial *S. fuscus* are used to form memories, but the absence of other sensory stimuli (i.e., chemical and physical) may interfere in memory formation and retention time.

The results presented suggest a difference between the SCG and DCG in the progression of agonistic displays, whereby different phases of the experimental procedure showed 10 days as an important period in which fewer agonistic displays occurred. Understanding the role of memory in potential agonistic encounters has major implications for elucidating social dynamics in fish (Francis, 1983). Studies involving recognition abilities have critical applications for understanding population structure and could help develop protocols for species conservation (Griffiths, 2003). We showed that *S. fuscus* retain a visual memory of the familiar opponent for at least 5 days, but in other contexts (appetitive and aversive place conditioning) retention goes beyond 15 days. For future studies involving the cognitive ability of reef fish, it is important to determine the relationship between the type of memory and retention time, as well as its effects on the dynamics of reef environments.

## Data Availability Statement

The raw data supporting the conclusions of this article will be made available by the authors, without undue reservation.

## Ethics Statement

The animal study was reviewed and approved by Animal Ethics Committee from Universidade Federal do Rio Grande do Norte (CEUA 041/2016).

## Author Contributions

AL and MS contributed to conception and design of the study. MS, JS, and HA-S organized the database. HA-S performed the statistical analysis. MS wrote the first draft of the manuscript. AL, MS, HA-S, and JS wrote sections of the manuscript. All authors contributed to the article and approved the submitted version.

## Conflict of Interest

The authors declare that the research was conducted in the absence of any commercial or financial relationships that could be construed as a potential conflict of interest.

## Publisher’s Note

All claims expressed in this article are solely those of the authors and do not necessarily represent those of their affiliated organizations, or those of the publisher, the editors and the reviewers. Any product that may be evaluated in this article, or claim that may be made by its manufacturer, is not guaranteed or endorsed by the publisher.
